# Improving speech intelligibility in noise and spatial perception: the critical role of hearing aid microphone position

**DOI:** 10.3389/fnins.2024.1475122

**Published:** 2024-10-21

**Authors:** Eyyup Kara, Nurşah Özal, Burcu Deniz, Talha Çögen, Rışvan Deniz, Kübra Aydın, Cenk Caba, Begüm Bahar Yılmaz

**Affiliations:** ^1^Department of Audiology, İstanbul University-Cerrahpaşa Faculty of Health Sciences, İstanbul, Türkiye; ^2^Department of Audiology, İstanbul University-Cerrahpaşa Institute of Graduate Studies, İstanbul, Türkiye; ^3^Department of Audiology, Eskişehir Osmangazi University Faculty of Health Sciences, Eskişehir, Türkiye; ^4^Department of Audiology, Koç University-Hospital, İstanbul, Türkiye; ^5^Ear-Teknik Hearing Aids, İstanbul, Türkiye; ^6^Department of Otorhinolaryngology, Başakşehir Çam and Sakura City Hospital, İstanbul, Türkiye

**Keywords:** hearing aid, speech intelligibility, auditory localization, hearing aid satisfaction, microphone location

## Abstract

**Introduction:**

Hearing aid (HA) manufacturers have introduced behind-the-ear (BTE) models where the microphone is positioned in the ear canal, which could impact auditory performance by distorting the pinna’s acoustic cues. This study aimed to compare two different BTE HAs with varying microphone positions: the receiver in the ear (RITE) and the transducer in the ear (TIE).

**Methods:**

The study involved 10 participants who had never used HAs before. They used both RITE and TIE HAs bilaterally for 3 weeks. Auditory performance was assessed through free field hearing assessments (hearing thresholds, speech recognition threshold, and speech discrimination score), the Turkish Matrix Sentence Test (TURMatrix), a sound localization test, and the Satisfaction with Amplification in Daily Living (SADL) questionnaire.

**Results:**

There was no significant difference between TIE and RITE in the free field hearing assessments. However, TIE outperformed RITE in non-adaptive TURMatrix scores in quiet, adaptive TURMatrix scores in noise, and sound localization accuracy at various angles. SADL sub-scores (Positive Effect, Service and Cost, and Personal Image) and overall satisfaction scores were significantly better for TIE.

**Discussion:**

The microphone position in HAs can influence auditory performance. This study demonstrated that TIE provided better speech intelligibility, localization accuracy, and user satisfaction compared to RITE.

## 1 Introduction

Hearing aids (HAs) are the primary solution for managing hearing loss that cannot be treated medically or surgically (Arlinger, 2003; Moore, 2003). One crucial consideration when selecting a HA is the device style (Davidson et al., 2022). HAs are broadly categorized into three types, each with different styles: behind-the-ear (BTE), in-the-ear (ITE), and in-the-canal (ITC). Factors such as the patient’s degree and configuration of hearing loss, ear canal anatomy, age, and daily needs determine the most appropriate HA style. Recent market analyses indicate that users most commonly use BTE HAs (64%), with the receiver in-the-ear (RITE) being the most popular BTE style (Picou et al., 2022). However, cosmetic concerns significantly influence the decision to use HAs, with many users preferring smaller, less visible devices (Prakash et al., 2022). Consequently, there has been a growing interest in HA styles that offer both cosmetic appeal and functional benefits.

**Figure 1:**
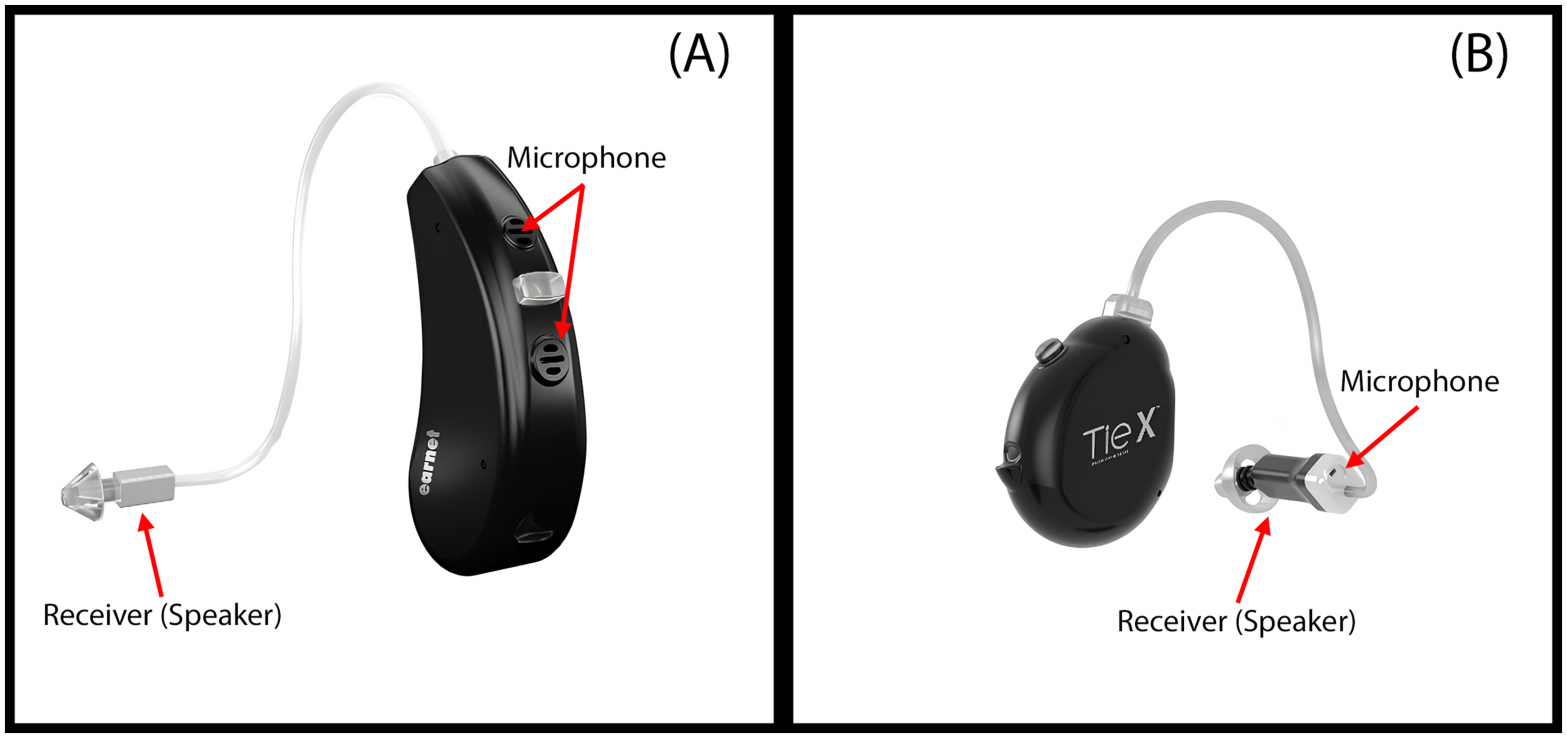
(A) Receiver-in-the-Ear (RITE) hearing aid: the microphone is located on the shell, while the receiver is positioned at the ear canal level. (B) Transducer-in-the-Ear (TIE) hearing aid: both the microphone and the receiver are located at the ear canal level.

There are two relatively new styles of BTE that differ depending on the location of the receiver (speaker): receiver in the aid (RIA or open fit) and receiver in the ear/canal (RITE/RIC) (Mondelli et al., 2015). RITE uses a flexible adapter, providing specialized amplification for hearing loss in the mid and high frequencies. On the other hand, ITE and ITC types are smaller devices that are suitable for individuals with mild to severe hearing loss, and the parts of the device are placed in a shell. The main difference between the two devices is that the ITC (or ITE) microphone is located at the level of the ear canal. Additionally, while ITC (or ITE) style has a single microphone, BTE style have two or more microphones on the front and back. The BTE (RITE) style is illustrated in Figure 1A.

Traditional BTE HAs position the microphones behind the ear, which may not capture the natural acoustic cues provided by the pinna and concha. This positioning can lead to suboptimal sound quality and artificial sound perception, potentially affecting speech intelligibility and localization abilities (Boyd et al., 2012). Studies have shown that BTE devices can distort high-frequency pinna cues, negatively impacting the perception of external sounds (Boyd et al., 2012; Denk et al., 2018). Conversely, ITE and ITC devices, with microphones located at the ear canal entrance, preserve these natural spectral cues, potentially enhancing sound localization and externalization (Byrne and Noble, 1998; Durin et al., 2014).

Several studies have compared the acoustic and perceptual differences between HA styles. Fortune (1997) suggested that ITE devices, by taking advantage of the pinna due to microphone location, are more advantageous in terms of directionality and speech intelligibility than BTE devices. Durin et al. (2014) conducted acoustic analyses of five HA models and found that Invisible-In-The-Canal (IIC) devices produced spectral cues most similar to unaided ears, while BTE devices showed the most significant deviations. Ricketts et al. (2001) reported that ITE devices provided better speech intelligibility in noise compared to BTE devices in omnidirectional mode. Similarly, Van den Bogaert et al. (2011) found that front-back localization performance was worse with BTE devices using omnidirectional microphones compared to ITC and in-the-pinna (remote-microphone) styles.

Localization ability can be affected by the HA model, microphone placement, type of stimuli, environmental conditions such as reverberation and noise, and the hearing abilities of individuals. BTE microphone position has been reported to have a 2–3 dB negative impact on signal-to-noise ratio (SNR) for lateral noise sources and frontally presented speech (Festen and Plomp, 1986). Additionally, BTE microphones are less effective at capturing directional cues, which are essential for accurate sound localization (Denk et al., 2018). However, findings across studies are not entirely consistent. Cox and Risberg (1986) found similar speech intelligibility between ITE and BTE devices, while Stone et al. (2023) reported comparable speech clarity ratings between RITE and ITE styles. Noble and Byrne (1990) observed that localization performance with ITC devices was worse than unaided performance, suggesting that device occlusion and individual variability may play roles.

Emerging HA designs aim to combine the discreetness of smaller devices with the technological capabilities of BTEs. One such design is the Transducer-in-the-Ear (TIE), which positions both the microphone and receiver within the ear canal while housing other components behind the ear. This configuration is comparable to ITE or ITC devices in terms of microphone placement but maintains the form factor of a BTE. The TIE design potentially offers the benefits of natural spectral cue preservation while accommodating various degrees of hearing loss. A similar design, called the Microphone and Receiver-In-Ear (M&RIE) HA, includes an additional microphone in the receiver of an otherwise RITE device. Groth (2022) evaluated localization in adults with normal hearing and mild hearing loss using the M&RIE HA, which includes an additional microphone in the receiver. Normal-hearing listeners made fewer front-back and overall localization errors with M&RIE compared to omnidirectional settings, mimicking pinna compensation. In those with hearing loss, significant improvements in localization and sound quality were observed only with M&RIE.

Understanding how microphone placement affects speech intelligibility, localization accuracy, and user satisfaction is crucial for optimizing HA design and fitting strategies. Since the main purpose of the HA is to facilitate communication by improving speech perception, it is important to evaluate these outcomes (Aazh and Moore, 2007; Gazia et al., 2020). User satisfaction is influenced by factors such as daily HA use time, previous device experience, self-perceived hearing difficulty, HA model, cost, and processing type (Uriarte et al., 2005). Studies have reported greater satisfaction with smaller HAs (Kochkin, 2000; Baumfield and Dillon, 2001), although age may also be a factor, with older HA users tending to report less satisfaction than younger individuals (Genç et al., 2018).

This study aimed to compare speech intelligibility, localization accuracy, and device satisfaction between TIE and RITE HAs with different microphone positions. We hypothesized that positioning the microphone at the entrance to the ear canal, as in the TIE device, would result in better speech understanding scores, improved localization accuracy, and higher satisfaction compared to the RITE device.

## 2 Materials and methods

Ethical approval of the study was received from the Clinical Research Ethics Committee (Approval No. 83045809–604.01.01). Participants provided written consent after being informed about the study. The study was conducted in accordance with the principles outlined in the Declaration of Helsinki.

### 2.1 Participants

An *a priori* power analysis was conducted using G*Power version 3.1.9.7 software (Faul et al., 2007) to determine the appropriate sample size, using effect sizes (*f* = 0.25 and d = 0.5) suggested by Cohen (1988), with a power level of 0.80 and an alpha level of 0.05. The analysis was performed for repeated-measures ANOVA and paired-samples t-tests. Assumptions made during the power analysis included moderate effect sizes, equal variances, normal distribution of the data, and sphericity for ANOVA. The larger sample size was selected as the minimum required sample size. Results indicated that at least 10 participants would provide sufficient power to detect moderate effects in the planned statistical tests.

Participants were included in the study if they had normal otoscopic examination and tympanometry findings, bilateral symmetrical sloping mild to moderate sensorineural hearing loss, and no cognitive or psychological disorders. Participants underwent comprehensive audiological assessments. Anamnesis and otoscopic examination were performed to evaluate medical history and ear health. Tympanometry and acoustic reflex thresholds were assessed using a middle ear analyzer with a 226 Hz probe tone to evaluate middle ear function. Pure-tone audiometry was conducted to measure air and bone conduction thresholds using an audiometer (Otometrics Astera, GN Otometrics, Taastrup, Denmark) with ER-3A insert earphones and a Radioear B-71 bone vibrator. Hearing loss was classified according to the guidelines of the American Speech-Language-Hearing Association (Clark, 1981). Additionally, speech audiometry was conducted to determine the Speech Reception Threshold (SRT) using the “Turkish Phonetically Balanced Word List,” and the Speech Discrimination Score (SDS) was measured using the “Turkish Phonetically Balanced Monosyllabic Word List” at the participant’s most comfortable listening level.

A total of 10 participants (7 females, 3 males) aged between 34 and 72 years (mean ± SD: 55.3 ± 14.74 years) with bilateral symmetrical sloping mild to moderate sensorineural hearing loss were included in the study.

### 2.2 Procedure

Two HA models with equivalent technical features and performance were used to compare the effects of microphone placement and design:

- Receiver-In-The-Ear (RITE) HAs: Bilateral devices with two microphones located behind the ear (Earnet Nano 3 RITE, M power receiver, Ear-Technic, Istanbul, Türkiye) (Figure 1A).
- Transducer-In-the-Ear (TIE) HAs: Bilateral devices with a single microphone and receiver located at the ear canal level (Earnet Modular S TIE X, M power receiver, Ear-Technic, Istanbul, Türkiye) (Figure 1B).

The term “transducer” refers to any device that converts one form of energy into another. In the context of HAs, both the microphone and receiver (speaker) are transducers: the microphone converts sound waves into electrical signals, while the receiver converts those electrical signals back into sound. In RITE HAs, only the receiver is placed in the ear canal, while the microphones remain behind the ear. In TIE devices, both transducers, the microphone and the receiver, are positioned within the ear canal.

#### 2.2.1 Randomization and adaptation period

Participants were randomly assigned to use each HA model in a counterbalanced order to prevent learning effects from influencing the results. Each participant used one HA model for a three-week adaptation period before switching to the other model for an additional three weeks. This approach ensured participants had ample time to acclimate to both devices.

#### 2.2.2 Hearing aid fitting

Both HAs were programmed using the National Acoustic Laboratories’ Non-Linear version 2 (NAL-NL2) prescription algorithm to ensure a standardized fitting. NAL-NL2 aims to maximize speech intelligibility while maintaining comfortable loudness (Keidser et al., 2011). Using the same algorithm minimized variability, allowing for a more accurate comparison of the devices’ performance.

To isolate the effects of physical design and microphone placement, additional features were standardized: noise reduction was disabled, and microphones were set to omnidirectional mode, eliminating influences from noise management and directional processing (Ricketts and Hornsby, 2005; Van den Bogaert et al., 2006).

Real-ear measurements were conducted using the Aurical FreeFit system (GN Otometrics) to verify that the prescribed amplification matched the NAL-NL2 targets. These measurements included assessing the natural ear canal resonance through the Real Ear Unaided Response (REUR), evaluating the acoustic effects of the HA shell or earmold when inserted but turned off using the Real Ear Occluded Response (REOR), and measuring the actual amplification provided by the HA when turned on via the Real Ear Aided Response (REAR). Ensuring that both HAs met the NAL-NL2 targets allowed us to attribute any observed performance differences to the devices themselves rather than fitting discrepancies (Jorgensen, 2016).

### 2.3 Assessments

After each three-week adaptation period, participants underwent a series of assessments to evaluate the performance of each HA model. These assessments included free-field aided pure-tone audiometry, speech audiometry, speech intelligibility testing using the Turkish Matrix Sentence Test (TURMatrix), a localization test, and the Satisfaction with Amplification in Daily Life (SADL).

#### 2.3.1 Free-field aided pure-tone audiometry

Free-field aided pure-tone audiometry was conducted to measure participants’ aided hearing thresholds at 500 Hz, 1,000 Hz, 2000 Hz, and 4,000 Hz, frequencies that are critical for speech understanding. Participants were seated 1 meter in front of a loudspeaker positioned at 0° azimuth within a sound-treated room to minimize ambient noise interference. A frequency-modulated (FM) stimulus was employed to prevent standing waves and ensure accurate threshold measurements. The Average Aided Threshold (AAT) was calculated by averaging the thresholds across the four frequencies, providing a single metric to compare the overall amplification provided by each HA (Kodera et al., 2016). This method ensured that both HAs were fitted according to similar prescription guidelines, thereby minimizing the impact of fitting differences on the results.

#### 2.3.2 Speech audiometry

Speech audiometry was used to assess participants’ speech perception abilities with each HA model. Two key measures were included: the Speech Reception Threshold (SRT) and the Speech Discrimination Score (SDS). The SRT was determined using the “Turkish Phonetically Balanced Word List,” with words presented through a loudspeaker at 0° azimuth. The SRT represents the lowest intensity level at which participants can correctly repeat 50% of the presented words. The SDS was measured using the “Turkish Phonetically Balanced Monosyllabic Word List” at the participant’s most comfortable listening level, and it reflects the percentage of words correctly identified, providing an indication of speech clarity.

#### 2.3.3 Speech intelligibility with Turkish Matrix Sentence Test (TURMatrix)

Speech intelligibility was assessed using the Turkish Matrix Sentence Test (TURMatrix), a standardized tool designed to evaluate speech recognition performance in various listening conditions for Turkish-speaking individuals (Zokoll et al., 2015). The TURMatrix comprises sentences constructed from a fixed syntactic structure of five words: name, numeral, adjective, object, and verb. These words are randomly selected from a 50-word base matrix, resulting in 10 sentences per test list that are syntactically uniform but semantically unpredictable.

In this study, both non-adaptive and adaptive TURMatrix protocols were employed to comprehensively evaluate participants’ speech recognition abilities:

Non-Adaptive TURMatrix (Test in Quiet): This test was conducted at a fixed presentation level of 65 dB Sound Pressure Level (SPL). Participants listened to sentences presented through a loudspeaker and were instructed to repeat each sentence verbatim. The percentage of correctly identified words was calculated to determine the speech intelligibility in quiet. Each correctly repeated word was awarded one point, with a maximum possible score of 50 per test list. Each participant was administered two test lists, consisting of a total of 20 sentences, and the percentage of correctly identified words was then calculated to determine speech intelligibility.

Adaptive TURMatrix (Test in Spatially Separated Noise): This test aimed to determine the Speech Reception Threshold (SRT), defined as the signal-to-noise ratio (SNR) at which participants correctly recognized 50% of the speech material. In this protocol, a constant background noise matching the long-term average speech spectrum was presented at 65 dB SPL. The speech signal level varied adaptively based on the participant’s responses, following a one-up, one-down procedure. The SRT and SNR were calculated based on the adaptive tracking of speech levels in relation to the fixed noise level.

Participants were seated in the center of the room, equidistant (1 meter) from both the front and rear loudspeakers. The front loudspeaker, positioned at 0° azimuth, delivered the speech signals, while the rear loudspeaker, positioned at 180° azimuth, emitted the background noise during the adaptive test.

Prior to formal testing, participants completed a brief training session using two practice sentences to familiarize themselves with the test procedure. Both the adaptive and non-adaptive protocols were administered under all three conditions: without a device, with RITE HAs, and with TIE HAs. All testing was conducted in a soundproof room to eliminate ambient noise interference. These protocols were employed with the aim of thoroughly comparing participants’ hearing abilities both in quiet and in noise, across different HA conditions. The TURMatrix is designed to simulate everyday listening situations, making it a valuable tool for assessing the practical benefits of HAs (Zokoll et al., 2015).

#### 2.3.4 Localization test

Localization abilities were assessed in a free-field environment within a sound-proof room. The test aimed to evaluate participants’ ability to accurately identify the direction of sound sources presented from various azimuthal positions.

The localization test utilized an array of eight loudspeakers, positioned horizontally at ear level in a circular arrangement around the participant at 45° intervals, covering azimuths from 0° to 315° (i.e., 0°, 45°, 90°, 135°, 180°, 225°, 270°, and 315°). Each loudspeaker was placed at a distance of 1 meter from the participant, ensuring equal sound pressure levels at the listening position. The system was calibrated using a sound level meter (Model 824, Larson-Davis) to ensure consistent output levels across all loudspeakers.

A frequency-modulated (FM) pure tone centered at 1 kHz, with a modulation depth of ±10% and a modulation rate of 5 Hz, was used as the test signal. The signal was presented at 65 dB SPL for a duration of approximately 3 s per stimulus.

The 1 kHz frequency was selected because it falls within the range of peak human auditory sensitivity, providing an optimal balance for testing localization abilities without favoring low or high-frequency hearing (Oxenham, 2018). Frequency modulation was employed to minimize the formation of standing waves in the testing environment. Standing waves can occur when pure tones reflect off room surfaces, leading to areas of constructive and destructive interference that create uneven sound pressure levels (Kuttruff and Vorländer, 2024). While more pronounced at lower frequencies, standing waves at 1 kHz can still affect the uniformity of the sound field. By slightly varying the frequency over time, the FM signal reduces the likelihood of standing wave patterns, ensuring a more consistent and uniform sound field throughout the room. Additionally, the FM tone’s distinctive modulation makes it easily recognizable and distinguishable, aiding participants in accurately identifying and localizing the test signal.

Prior to formal testing, participants underwent a training session to familiarize themselves with the test procedure and the spatial arrangement of the loudspeakers. During training, two practice stimuli were presented from each of the eight loudspeaker positions, and participants received feedback on their responses. For the actual test, a total of 40 stimuli were presented in a randomized order, with each loudspeaker delivering five stimuli. Participants were instructed to remain seated at the center of the loudspeaker array and to keep their heads oriented forward, avoiding head movements during stimulus presentation to ensure that localization judgments were based solely on auditory cues. At the onset of each trial, a single stimulus was presented from one of the eight loudspeakers. Participants were asked to identify the direction of the sound source by verbally indicating the corresponding azimuthal angle by pointing to a diagram provided on a response sheet. The diagram depicted the loudspeaker positions labeled with their respective angles, serving as a visual aid to facilitate accurate responses. The primary outcome measure was the percentage of correctly identified sound source locations for each participant. The localization test was repeated for all three conditions: without a device, with RITE HAs, and with TIE HAs. Ultimately, the test assessed the ability to identify the direction of sound sources, which may be influenced by microphone placement and the design of the HAs (Hassager et al., 2017).

#### 2.3.5 Hearing aid satisfaction

Participants’ satisfaction with each HA (RITE and TIE) was assessed using the Turkish version of the Satisfaction with Amplification in Daily Life (SADL) scale, a validated tool for measuring HA satisfaction across various dimensions (Cox and Alexander, 1999; Genç et al., 2018). The SADL provides a global score and covers four subscales: Positive Effect (psychoacoustic improvement and psychological impact), Service and Cost (value for money and provider confidence), Negative Features (issues like background noise and feedback), and Personal Image (concerns about appearance and stigma). The SADL consists of 15 items rated on a 7-point Likert scale, from “Not at All” to “Tremendously.” Global and subscale scores are calculated by averaging the corresponding item scores, with higher scores indicating greater satisfaction.

Participants completed the SADL after three weeks of using each HA, with the order of device usage randomized. The questionnaire was self-administered on paper in a quiet room, with a researcher available for assistance.

### 2.4 Statistical analysis

Statistical analyses were conducted using R Statistical Analysis Software Version 4.4.1 (R Core Team, 2024). Continuous variables are expressed using mean, standard deviation and 95% confidence intervals. The localization test scores are displayed as correct percentage. The Shapiro–Wilk test was used to assess the normality of data distributions. All variables met the assumption of normality (*p* > 0.05).

Repeated Measures Analysis of Variance (ANOVA) was employed to compare the localization test results and the adaptive and non-adaptive TURMatrix results under three conditions: without any device, with RITE HA, and with TIE HA. Mauchly’s Test of Sphericity was conducted to assess the assumption of sphericity. When the sphericity assumption was violated, the Greenhouse–Geisser correction was applied. *Post hoc* analyses were performed using Tukey’s Honestly Significant Difference (HSD) test to identify pairwise differences between conditions.

Paired sample t-tests were used to compare the AAT, SRT, SDS, and SADL scores of participants when using TIE versus RITE devices. Cronbach’s alpha was calculated to evaluate the internal consistency of the SADL questionnaire responses.

Effect sizes were calculated using partial eta squared (η^2^) for ANOVA analyses and Cohen’s d for t-tests to provide a measure of the practical significance of the findings. A significance level of *α* = 0.05 was used for all statistical tests. Adjustments for multiple comparisons were made using the Bonferroni correction where appropriate. All analyses were two-tailed, and 95% confidence intervals (CIs) were reported to provide precision estimates for the effect sizes.

## 3 Results

The study included 10 participants, consisting of 7 females and 3 males, with a mean age of 55.3 years (SD = 14.74). The Shapiro–Wilk test confirmed that the data used for statistical analysis followed a normal distribution (*p* > 0.05). Descriptive statistics for the participant demographics and test scores are presented in Tables 1, 2.

**Table 1:** Descriptive statistics and paired sample t-test results for measures across RITE and TIE conditions.

	df	*M*	SD	95% CI	*t*	*p*	*d*
Age		55.30	14.74	[44.75–65.85]			
AAT							
RITE	9	28.38	5.71	[24.29–32.46]	0.94	0.370	0.30
TIE		29.13	6.72	[24.32–33.93]			
SRT							
RITE	9	28.00	7.53	[22.61–33.39]	−0.43	0.678	−0.14
TIE		27.50	8.58				
SDS							
RITE	9	86.00	6.60	[81.28–90.72]	2.21	0.054	0.70
TIE		89.60	4.70	[86.24–92.96]			
SADL-Total							
RITE	9	3.75	0.56	[3.35–4.15]	3.33	0.009	1.05
TIE		4.32	0.97	[3.63–5.01]			
SADL-PE							
RITE	9	3.71	1.44	[2.69–4.74]	4.00	0.003	1.26
TIE		4.51	1.76	[3.25–5.78]			
SADL-NF							
RITE	9	4.30	0.95	[3.62–4.98]	−0.36	0.726	−0.11
TIE		4.20	1.07	[3.43–4.96]			
SADL-PI							
RITE	9	2.62	1.05	[1.87–3.37]	2.79	0.021	0.09
TIE		3.66	1.77	[2.4–4.93]			
SADL-S&C							
RITE	9	3.63	0.92	[2.97–4.29]	6.13	< 0.001	1.94
TIE		4.73	1.15	[3.91–5.55]			

AAT: Average Aided Threshold, SRT: Speech Reception Threshold, SDS: Speech Discrimination Score, RITE: Receiver in the Ear. TIE: Transducer in the Ear, SADL: Satisfaction with Amplification in Daily Living, PE: Positive Effect, NE: Negative Features. PI: Personal Image, S&C: Service & Cost.

**Table 2:** Repeated measures ANOVA results for TURMatrix and localization test measures across WD, RITE, and TIE conditions.

	Num df	Den df	*M*	SD	95% CI	*F*	*p*	Partial η^2^
Non-Adaptive TURMatrix								
WD	2	18	75.50	19.59	[61.48–89.52]	6.386	0.008	0.42
RITE			84.20	11.62	[75.89–92.51]			
TIE			89.40	11.69	[81.04–97.76]			
Adaptive TURMatrix								
WD	2	18	0.97	4.99	[−2.6–4.54]	8.178	0.003	0.48
RITE			−1.90	4.35	[−5.01–1.21]			
TIE			−4.72	5.03	[−8.32--1.12]			
Localization Test								
WD	2	18	37.50	24.30	[20.12–54.88]	5.774	0.012	0.39
RITE			41.25	20.45	[26.62–55.88]			
TIE			66.25	14.49	[55.88–76.62]			

TURMatrix: Turkish Matrix Sentence Test, WD: Without Device, RITE: Receiver-in-The-Ear, TIE: Transducer-in-the-Ear.

### 3.1 Free field aided hearing assessment

Paired samples t-tests was performed to evaluate whether there was a difference between TIE and RITE conditions in terms of AAT, SRT, and SDS scores. The results indicated no significant difference between the conditions for AAT, t(9) = 0.94, *p* = 0.370, SRT, t(9) = −0.43, *p* = 0.678, and SDS, t(9) = 2.21, *p* = 0.054. The details of the comparisons are presented in Table 1.

### 3.2 Speech intelligibility with Turkish Matrix Sentence Test (TURMatrix)

**Figure 2:**
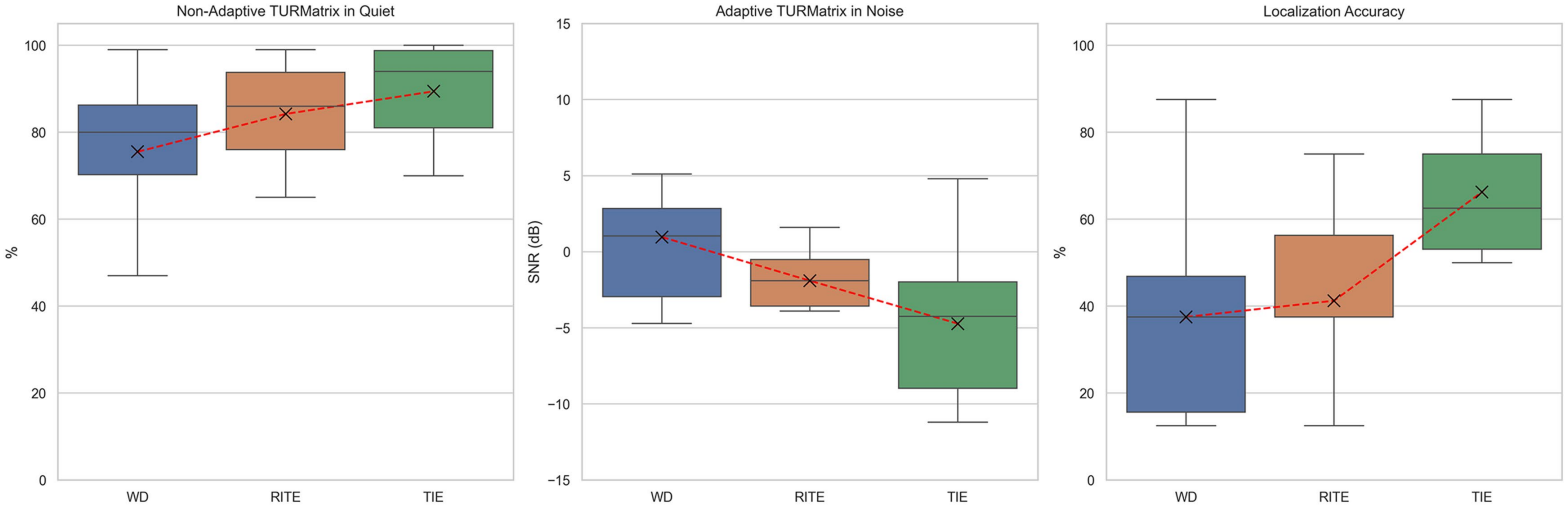
Non-adaptive (quiet) and adaptive (noise) Turkish Matrix Sentence Test (TURMatrix) results, and localization accuracy for Receiver-in-the-Ear (RITE), Transducer-in-the-Ear (TIE), and without device (WD) conditions.

A repeated-measures ANOVA was performed to evaluate the effect of hearing condition (without device, RITE, and TIE) on Non-Adaptive TURMatrix and Adaptive TURMatrix performance. The means and standard deviations for both tests are shown in Table 2, with the data visually represented in Figure 2.

Mauchly’s test indicated that the assumption of sphericity had been met for both the Non-Adaptive TURMatrix (χ^2^(2) = 0.583, *p* = 0.115) and the Adaptive TURMatrix (χ^2^(2) = 0.967, *p* = 0.875), so no corrections were applied to the degrees of freedom. The effect of hearing condition on Non-Adaptive TURMatrix was significant at the 0.05 level, *F*(2, 18) = 6.386, *p* = 0.008, partial η^2^ = 0.42, and the effect of hearing condition on Adaptive TURMatrix was also significant, F(2, 18) = 8.178, *p* = 0.003, partial η^2^ = 0.48.

Post-hoc pairwise comparisons with a Tukey HSD adjustment indicated that there was a significant difference between the Non-Adaptive TURMatrix performance in the TIE and Without Device conditions (*p* = 0.009), but there was no significant difference between the TIE and RITE conditions (*p* = 0.230) or between the Without Device and RITE conditions (*p* = 0.245). For the Adaptive TURMatrix, post-hoc pairwise comparisons revealed a significant difference between the TIE and Without Device conditions (*p* = 0.011), but no significant difference between the TIE and RITE conditions (*p* = 0.171) or between the Without Device and RITE conditions (*p* = 0.119).

### 3.3 Localization test

A repeated-measures ANOVA was performed to evaluate the effect of hearing condition (without device, RITE, and TIE) on Localization Test performance. The means and standard deviations are presented in Table 2, with the data also visually represented in Figure 2.

Mauchly’s test indicated that the assumption of sphericity had been violated (χ^2^(2) = 0.364, *p* = 0.018), and therefore degrees of freedom were corrected using Greenhouse–Geisser estimates of sphericity (*ε* = 0.637). The effect of hearing condition on Localization Test performance was significant at the *α* = 0.05 level, *F*(1.27, 11.45) = 5.774, *p* = 0.012, partial η^2^ = 0.39.

**Figure 3:**
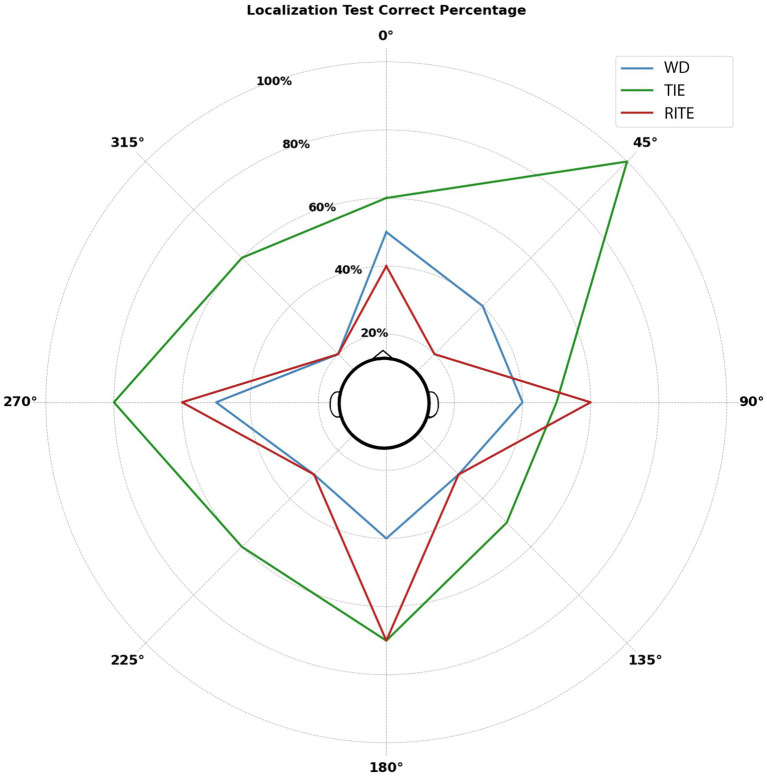
Correct localization percentages by azimuth for each condition: Without Device (WD), Receiver-in-the-Ear (RITE), and Transducer-in-the-Ear (TIE).

Post-hoc pairwise comparisons with a Tukey HSD adjustment indicated that there was a significant difference between the Localization Test performance in the TIE and Without Device conditions (*p* = 0.041), as well as between the TIE and RITE conditions (*p* = 0.001). However, no significant difference was found between the Without Device and RITE conditions (*p* = 0.945). The correct response percentages for each azimuthal angle used in the test are shown in Figure 3 using a radar plot for each hearing condition.

### 3.4 Hearing aid satisfaction (SADL)

**Figure 4:**
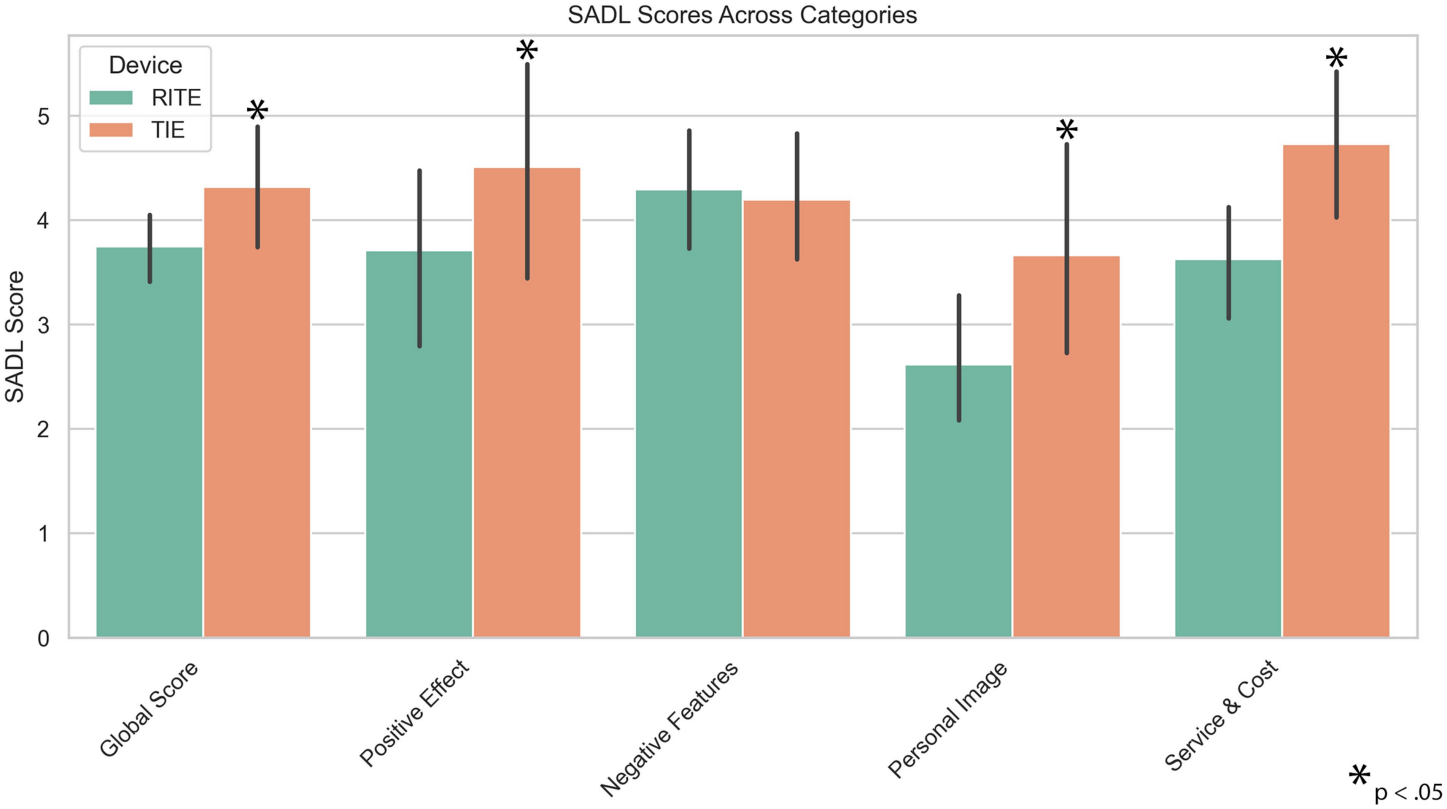
Satisfaction with Amplification in Daily Living (SADL) results for Receiver-in-the-Ear (RITE) and Transducer-in-the-Ear (TIE) hearing aids.

The SADL scale demonstrated good internal consistency, with a Cronbach’s alpha of 0.828. A paired samples t-test was performed to evaluate whether there was a difference between TIE and RITE conditions in terms of SADL-Global, Positive Effect (PE), Negative Features (NF), Personal Image (PI), and Service & Cost (S&C) scores. The results indicated that the TIE condition showed significantly higher scores than the RITE condition for SADL-Global, t(9) = 3.33, *p* = 0.009, and Positive Effect (PE), t(9) = 4.00, *p* = 0.003. Similarly, TIE had significantly higher scores than RITE for Personal Image (PI), t(9) = 2.79, *p* = 0.021, and Service & Cost (S&C), t(9) = 6.13, *p* < 0.001. However, no significant difference was found between TIE and RITE conditions for Negative Features (NF), t(9) = −0.36, *p* = 0.726. These comparisons are detailed in Table 1, with the data visually represented in Figure 4.

## 4 Discussion

The effect of microphone position on speech intelligibility and localization accuracy was investigated in this study. The results showed that the TIE provided better speech intelligibility (in both quiet and noise) and higher localization accuracy compared to the RITE, although there was no significant difference in average aided hearing thresholds. Additionally, participants reported higher satisfaction with the TIE than with the RITE.

Positioning the HA microphone in the ear canal gives the user greater access to the spectral cues of the pinna and concha, which could theoretically aid in sound localization and perception of externality (Boyd et al., 2012). A study comparing the acoustic analysis of five different HA models showed that the most similar result to bare ear recordings was with the invisible in the canal (IIC), while the most different result was with the BTE device (Durin et al., 2014). Another study found that the BTE’s frequency response and microphone location had a negative impact on people with normal hearing, distorting their perception of external sounds by distorting high-frequency pinna cues (Boyd et al., 2012). The commercial HA model most similar to the TIE style is the microphone and receiver in ear (M&RIE). In a non-peer-reviewed study performed with M&RIE, the localization ability of five adults with normal hearing and ten adults with mild hearing loss was evaluated at 30-degree angles (Groth, 2022). The results showed that listeners with normal hearing experienced fewer front-back and overall localization errors with M&RIE compared to omnidirectional, providing similar benefits to pinna compensation. In patients with hearing loss, significantly better results were observed only with M&RIE. Additionally, M&RIE was found to have superior overall and spatial sound quality. The M&RIE comprises three microphones: two situated on the device and one positioned behind the receiver in the ear canal. Conversely, a TIE is comparable to an ITE or ITC device in that it just has one microphone placed in the ear canal.

Since the main purpose of the HA is to facilitate the communication process by improving speech intelligibility, it is important to test speech perception. There are few studies that investigate the effect of HA style on speech intelligibility. One of the previous studies compared speech intelligibility with ITE and BTE in nine participants with mild to moderate hearing loss. It was reported in the study that similar speech intelligibility was achieved with ITE and BTE devices (Cox and Risberg, 1986). In a recent study, RITE and ITE styles were rated similarly for speech clarity (Stone et al., 2023). On the other hand, Ricketts et al. (2001) reported that BTE performance in omnidirectional mode was lower than that of the ITE device (Ricketts et al., 2001). In our study, we observed worse speech intelligibility scores in quiet and noise with the RITE device in omnidirectional mode.

Localization ability can be affected by the HA model, type of stimuli, environmental conditions such as reverberation and noise, and the hearing abilities of individuals. BTE microphone position has been reported to have a 2–3 dB negative impact on SNR for lateral noise sources and frontally presented speech (Festen and Plomp, 1986). Bogaert et al. found that there was no difference in left–right localization performance with different HA styles (ITC, BTE, and in-the-pinna), and that the front-back localization performance of the BTE device using omnidirectional microphones was worse than other styles (Van den Bogaert et al., 2011). Additionally, a previous study reported that BTE microphones are quite poor at capturing directional cues (Denk et al., 2018). Other studies have reported different findings from previous studies. In another previous study investigating the effects of BTE, ITE, and ITC devices on horizontal and vertical localization, performance with only the ITC device was found to be worse than the performance of the same participants without the device (Noble and Byrne, 1990). Another study showed that, after a six-week trial, lateral and polar angle localization was not affected by HA style, and front-back reversal was lower in CIC than in BTE (Best et al., 2010). Byrne claimed that the ITE advantage helps performance in a test in which all sounds are presented at the same intensity, and that this advantage may not occur in real life (Byrne and Noble, 1998). However, since most studies involve measurements made on an artificial head, these results may not reflect the results of individuals or vice versa. This study did not evaluate real-world performance or perform acoustic analyzes in the artificial head. Therefore, the localization test performed at the same intensity in a quiet booth may have resulted in a TIE advantage.

Although verification of the HA can be achieved through real ear measurements and functional gain measurements, it is necessary to evaluate the user’s satisfaction and benefit after fitting (Aazh and Moore, 2007; Gazia et al., 2020). Daily HA use time, previous device use experience, self-perceived hearing difficulty, HA model, cost of HA and processing type used in the device are factors that affect satisfaction (Uriarte et al., 2005). Due to the limited number of participants and wide age range in our study, we did not investigate the effect of age on the results. However, it has been reported that age is also an important factor for satisfaction, with HA users tending to report less satisfaction than younger people (Genç et al., 2018). Wang et al. (2022) reported that the International Outcome Inventory for Hearing Aids (IOI-HA) scores in participants using three different HA styles were BTE, RIC and ITE, from low to high, respectively (Wang et al., 2022). Similarly, Uriarte et al. (2005) found that a significant effect of HA style on global satisfaction, personal image, and positive effect. In the study, Bonferroni comparison revealed that BTE and ITE provided significantly higher satisfaction for the positive effect subscale, and ITC for the personal image subscale. However, there was no significant effect of HA style on the Service & Cost and Negative Features subscales. In our study, the negative features subscale was not different for RITE and TIE HAs. The findings of the current study may be explained by previous studies reporting greater satisfaction with smaller HAs (Kochkin, 2000; Baumfield and Dillon, 2001).

### 4.1 Limitations

This study offers valuable insights into the effects of microphone placement on speech intelligibility and localization accuracy in HAs. However, as with any research, there are a few limitations that warrant consideration.

First, although the study included ten participants, which was sufficient to detect moderate effects as per the *a priori* power analysis, a larger sample size might enhance the generalizability of the findings. Future studies with more participants may provide more detailed insights, particularly in understanding subgroup differences, such as age-related effects.

Second, while the three-week adaptation period for each HA allowed participants to become accustomed to the devices, some research suggests that longer periods could yield further improvements in speech understanding (Saji et al., 2017). Future studies could explore the potential benefits of longer adaptation periods to assess more subtle effects of different HA models.

Third, although the study was conducted in a controlled, sound-treated environment to ensure consistency, real-world conditions might offer additional insights. Incorporating everyday listening environments in future research would help in understanding the practical implications of microphone placement in daily life.

Lastly, while this study primarily focused on behavioral outcomes, future research could also incorporate detailed acoustic analyses, such as evaluating how behind-the-ear components influence pinna cues. This would provide a deeper understanding of the acoustic factors contributing to the observed effects.

## 5 Conclusion

Participants with moderate sloping hearing loss using the TIE showed better auditory performance than RITE when considering improvement in speech recognition in situations of silence and noise, localization accuracy, and amplification satisfaction. The benefits of placing the TIE microphone in the ear canal can be achieved if the degree and type of hearing loss are acceptable.

## Data Availability

The raw data supporting the conclusions of this article will be made available by the authors, without undue reservation.

## References

[ref1] Aazh H. Moore B. C. (2007). The value of routine real ear measurement of the gain of digital hearing aids. J. Am. Acad. Audiol. 18, 653–664. doi: 10.3766/jaaa.18.8.3 18326152

[ref2] Arlinger S. (2003). Negative consequences of uncorrected hearing loss-a review. Int. J. Audiol. 42 Suppl 2, 2S17–12S20. doi: 10.3109/14992020309074639, PMID: 12918624

[ref3] Baumfield A. Dillon H. (2001). Factors affecting the use and perceived benefit of ITE and BTE hearing aids. Br. J. Audiol. 35, 247–258. doi: 10.1080/00305364.2001.11745243 11694099

[ref4] Best V. Kalluri S. McLachlan S. Valentine S. Edwards B. Carlile S. (2010). A comparison of CIC and BTE hearing aids for three-dimensional localization of speech. Int. J. Audiol. 49, 723–732. doi: 10.3109/14992027.2010.484827, PMID: 20515424

[ref5] Boyd A. W. Whitmer W. M. Soraghan J. J. Akeroyd M. A. (2012). Auditory externalization in hearing-impaired listeners: the effect of pinna cues and number of talkers. J. Acoust. Soc. Am. 131:EL268-EL274. doi: 10.1121/1.3687015, PMID: 22423819 PMC3635013

[ref6] Byrne D. Noble W. (1998). Optimizing sound localization with hearing AIDS. Trends Amplif. 3, 51–73. doi: 10.1177/108471389800300202, PMID: 25425879 PMC4172152

[ref7] Clark J. G. (1981). Uses and abuses of hearing loss classification. ASHA 23, 493–500, PMID: 7052898

[ref8] Cohen J. (1988). Statistical power analysis for the behavioral sciences. New York, NY: Routledge Member of the Taylor and Francis Group.

[ref9] Cox R. M. Alexander G. C. (1999). Measuring satisfaction with amplification in daily life: the SADL scale. Ear Hear. 20, 306–320. doi: 10.1097/00003446-199908000-00004, PMID: 10466567

[ref10] Cox R. M. Risberg D. M. (1986). Comparison of in-the-ear and over-the-ear hearing aid fittings. J. Speech Hear. Disord. 51, 362–369. doi: 10.1044/jshd.5104.362 3773492

[ref11] Davidson A. Marrone N. Souza P. (2022). Hearing aid technology settings and speech-in-noise difficulties. Am. J. Audiol. 31, 21–31. doi: 10.1044/2021_aja-21-00176, PMID: 35133851 PMC9128736

[ref12] Denk F. Ewert S. D. Kollmeier B. (2018). Spectral directional cues captured by hearing device microphones in individual human ears. J. Acoust. Soc. Am. 144, 2072–2087. doi: 10.1121/1.5056173, PMID: 30404454

[ref13] Durin V. Carlile S. Guillon P. Best V. Kalluri S. (2014). Acoustic analysis of the directional information captured by five different hearing aid styles. J. Acoust. Soc. Am. 136, 818–828. doi: 10.1121/1.4883372, PMID: 25096115

[ref14] Faul F. Erdfelder E. Lang A.-G. Buchner A. (2007). G*power 3: a flexible statistical power analysis program for the social, behavioral, and biomedical sciences. Behav. Res. Methods 39, 175–191. doi: 10.3758/BF03193146, PMID: 17695343

[ref15] Festen J. M. Plomp R. (1986). Speech-reception threshold in noise with one and two hearing aids. J. Acoust. Soc. Am. 79, 465–471. doi: 10.1121/1.393534 3950200

[ref16] Fortune T. W. (1997). Real-ear polar patterns and aided directional sensitivity. J. Am. Acad. Audiol. 8, 119–131, PMID: 9101458

[ref17] Gazia F. Galletti B. Portelli D. Alberti G. Freni F. Bruno R.. (2020). Real ear measurement (REM) and auditory performances with open, tulip and double closed dome in patients using hearing aids. Eur. Arch. Otorrinolaringol. 277, 1289–1295. doi: 10.1007/s00405-020-05822-1, PMID: 32008077

[ref18] Genç M. Çildir B. Kaya M. (2018). Psychometric properties of the Turkish version of the satisfaction with amplification in daily living questionnaire in hearing aid users. J. Am. Acad. Audiol. 29, 898–908. doi: 10.3766/jaaa.17073, PMID: 30479262

[ref19] Groth J. (2022). An innovative RIE with microphone in the ear lets users “hear with their own ears”, [Online]. Canadian Audiol. Available at: https://canadianaudiologist.ca/resound-feature-2/

[ref20] Hassager H. G. Wiinberg A. Dau T. (2017). Effects of hearing-aid dynamic range compression on spatial perception in a reverberant environment. J. Acoust. Soc. Am. 141, 2556–2568. doi: 10.1121/1.4979783 28464692

[ref21] Jorgensen L. E. (2016). Verification and validation of hearing aids: opportunity not an obstacle. J Otol 11, 57–62. doi: 10.1016/j.joto.2016.05.001, PMID: 29937811 PMC6002586

[ref22] Keidser G. Dillon H. Flax M. Ching T. Brewer S. (2011). The NAL-NL2 prescription procedure. Audiol Res. 1:e24. doi: 10.4081/audiores.2011.e24, PMID: 26557309 PMC4627149

[ref23] Kochkin S. (2000). MarkeTrak V: consumer satisfaction revisited. Hearing J. 53, 38–40. doi: 10.1097/00025572-200001000-00005

[ref24] Kodera K. Hosoi H. Okamoto M. Manabe T. Kanda Y. Shiraishi K.. (2016). Guidelines for the evaluation of hearing aid fitting (2010). Auris Nasus Larynx 43, 217–228. doi: 10.1016/j.anl.2015.10.015, PMID: 26654157

[ref25] Kuttruff H. Vorländer M. (2024). Room acoustics. Boca Raton: CRC Press.

[ref26] Mondelli M. F. C. G. Garcia T. M. Hashimoto F. M. T. Rocha A. V. (2015). Open fitting: performance verification of receiver in the ear and receiver in the aid. Braz. J. Otorhinolaryngol. 81, 270–275. doi: 10.1016/j.bjorl.2014.08.013, PMID: 25382428 PMC9452248

[ref27] Moore B. C. (2003). Speech processing for the hearing-impaired: successes, failures, and implications for speech mechanisms. Speech Comm. 41, 81–91. doi: 10.1016/S0167-6393(02)00095-X

[ref28] Noble W. Byrne D. (1990). A comparison of different binaural hearing aid systems for sound localization in the horizontal and vertical planes. Br. J. Audiol. 24, 335–346. doi: 10.3109/03005369009076574, PMID: 2265304

[ref29] Oxenham A. J. (2018). How we hear: the perception and neural coding of sound. Annu. Rev. Psychol. 69, 27–50. doi: 10.1146/annurev-psych-122216-011635, PMID: 29035691 PMC5819010

[ref30] Picou E. M. Roberts R. A. Angley G. Ricketts T. A. (2022). Applying the hearing aid fitting standard to selection for adults. Semin. Hear. 43, 066–078. doi: 10.1055/s-0042-1748874, PMID: 35903077 PMC9325089

[ref31] Prakash P. Sreedhar A. Balan J. R. Varghese A. M. (2022). Benefit on daily listening with technological advancements: comparison of basic and premium category hearing aids. Eur. Arch. Otorrinolaringol. 279, 3179–3187. doi: 10.1007/s00405-021-07240-3, PMID: 35038028 PMC8762635

[ref32] R Core Team (2024). R: A language and environment for statistical computing. Vienna, Austria: R Foundation for Statistical Computing.

[ref33] Ricketts T. A. Hornsby B. W. (2005). Sound quality measures for speech in noise through a commercial hearing aid implementing digital noise reduction. J. Am. Acad. Audiol. 16, 270–277. doi: 10.3766/jaaa.16.5.2, PMID: 16119254

[ref34] Ricketts T. Lindley G. Henry P. (2001). Impact of compression and hearing aid style on directional hearing aid benefit and performance. Ear Hear. 22, 348–361. doi: 10.1097/00003446-200108000-00009, PMID: 11527041

[ref35] Saji J. S. Madikeri Mohan K. Rajashekar B. (2017). Influence of channel and ChannelFree™ processing technology on the vocal parameters in hearing-impaired individuals. Int. J. Dis. Hum. Dev. 16, 45–53. doi: 10.1515/ijdhd-2016-0021

[ref36] Stone M. A. Lough M. Kühnel V. Biggins A. E. Whiston H. Dillon H. (2023). Perceived sound quality of hearing aids with varying placements of microphone and receiver. Am. J. Audiol. 32, 135–149. doi: 10.1044/2022_aja-22-00061, PMID: 36580494 PMC10166191

[ref37] Uriarte M. Denzin L. Dunstan A. Sellars J. Hickson L. (2005). Measuring hearing aid outcomes using the satisfaction with amplification in daily life (SADL) questionnaire: Australian data. J. Am. Acad. Audiol. 16, 383–402. doi: 10.3766/jaaa.16.6.6 16178409

[ref38] Van den Bogaert T. Carette E. Wouters J. (2011). Sound source localization using hearing aids with microphones placed behind-the-ear, in-the-canal, and in-the-pinna. Int. J. Audiol. 50, 164–176. doi: 10.3109/14992027.2010.537376, PMID: 21208034

[ref39] Van den Bogaert T. Klasen T. J. Moonen M. Van Deun L. Wouters J. (2006). Horizontal localization with bilateral hearing aids: without is better than with. J. Acoust. Soc. Am. 119, 515–526. doi: 10.1121/1.2139653 16454305

[ref40] Wang X. Zheng Y. Liu Y. Lu J. Cui Z. Li Z. (2022). Effects of demographic, audiologic, and hearing-aid-related variables on the outcomes of using hearing aids. Eur. Arch. Otorrinolaringol. 279, 3857–3865. doi: 10.1007/s00405-021-07126-4 34725721

[ref41] Zokoll M. A. Fidan D. Türkyılmaz D. Hochmuth S. Ergenç İ. Sennaroğlu G.. (2015). Development and evaluation of the Turkish matrix sentence test. Int. J. Audiol. 54, 51–61. doi: 10.3109/14992027.2015.1074735, PMID: 26443486

